# Differential Effects of Sustained Manual Pressure Stimulation According to Site of Action

**DOI:** 10.3389/fnins.2019.00722

**Published:** 2019-07-17

**Authors:** Pavel Hok, Jaroslav Opavský, René Labounek, Miroslav Kutín, Martina Šlachtová, Zbyněk Tüdös, Petr Kaňovský, Petr Hluštík

**Affiliations:** ^1^Department of Neurology, University Hospital Olomouc, Olomouc, Czechia; ^2^Department of Neurology, Faculty of Medicine and Dentistry, Palacký University Olomouc, Olomouc, Czechia; ^3^Department of Physiotherapy, Faculty of Physical Culture, Palacký University Olomouc, Olomouc, Czechia; ^4^Department of Biomedical Engineering, University Hospital Olomouc, Olomouc, Czechia; ^5^KM KINEPRO PLUS s.r.o., Olomouc, Czechia; ^6^Department of Radiology, Faculty of Medicine and Dentistry, Palacký University Olomouc, Olomouc, Czechia; ^7^Department of Radiology, University Hospital Olomouc, Olomouc, Czechia

**Keywords:** magnetic resonance imaging, neurological rehabilitation, physical stimulation, sensorimotor cortex, brainstem

## Abstract

Sustained pressure stimulation of the body surface has been used in several physiotherapeutic techniques, such as reflex locomotion therapy. Clinical observations of global motor responses and subsequent motor behavioral changes after stimulation in certain sites suggest modulation of central sensorimotor control, however, the neuroanatomical correlates remain undescribed. We hypothesized that different body sites would specifically influence the sensorimotor system during the stimulation. We tested the hypothesis using functional magnetic resonance imaging (fMRI) in thirty healthy volunteers (mean age 24.2) scanned twice during intermittent manual pressure stimulation, once at the right lateral heel according to reflex locomotion therapy, and once at the right lateral ankle (control site). A flexible modeling approach with finite impulse response basis functions was employed since non-canonical hemodynamic response was expected. Subsequently, a clustering algorithm was used to separate areas with differential timecourses. Stimulation at both sites induced responses throughout the sensorimotor system that could be mostly separated into two anti-correlated subsystems with transient positive or negative signal change and rapid adaptation, although in heel stimulation, insulo-opercular cortices and pons showed sustained activation. In direct voxel-wise comparison, heel stimulation was associated with significantly higher activation levels in the contralateral primary motor cortex and decreased activation in the posterior parietal cortex. Thus, we demonstrate that the manual pressure stimulation affects multiple brain structures involved in motor control and the choice of stimulation site impacts the shape and amplitude of the blood oxygenation level-dependent response. We further discuss the relationship between the affected structures and behavioral changes after reflex locomotion therapy.

## Introduction

Neuronal plasticity is a key component in restoration of human motor function. Plastic changes can be induced via transient peripheral afferent stimulation (Powell et al., 1999). Outlasting modulatory effects in the sensorimotor cortex have been observed following sustained electrical (Chipchase et al., 2011), magnetic (Gallasch et al., 2015), and vibratory (Rosenkranz and Rothwell, 2003) stimulation. Peripheral pressure stimulation has been studied less extensively (Miura et al., 2013; Chung et al., 2014, 2015; Sanz-Esteban et al., 2018) despite the fact that it serves as a major component of clinical physiotherapeutic techniques, such as the “reflex locomotion” (Vojta, 1973; Vojta and Peters, 2007; Hok et al., 2017; Jung et al., 2017).

The technique, also known as Vojta method, uses sustained manual pressure stimulation of specific body surface areas to gradually evoke a stereotypic pattern of tonic muscle contractions in both sides of the neck, trunk, and limbs (Vojta, 1973). It has been speculated that the motor response is controlled by a brainstem region (Laufens et al., 1991; Hok et al., 2017), possibly related to the so-called central pattern generators that were discovered in vertebrate animals (Grillner and Wallén, 1985) and more recently became associated with human locomotion and postural control (Jahn et al., 2008; la Fougère et al., 2010; Takakusaki, 2013). Indeed, we have previously shown that heel stimulation according to Vojta specifically modulates subsequent motor task-related activation in the dorsal pons, medulla (presumably in the pontomedullary reticular formation, PMRF), and cerebellum (Hok et al., 2017). Nevertheless, there is limited knowledge of the immediate neurobiological correlates of the therapeutic stimulation and the resulting interaction between the somatosensory and motor system.

Previous imaging studies of pressure stimulation recently provided valuable, yet still incomplete picture of the central somatosensory processing (Hao et al., 2013; Miura et al., 2013; Chung et al., 2014, 2015; Sanz-Esteban et al., 2018). Miura et al. (2013) observed bilateral activation in the primary and secondary somatosensory cortices during short manual foot sole stimulation applied at the base of the toes over 5 s. Similar pattern has been observed during 30 s of 1-Hz sinusoidal pressure applied to the foot sole (Hao et al., 2013). Chung et al. (2015) described patterns of somatosensory activations during static sustained pressure stimulation of the index finger tip, providing imaging evidence for gradual adaptation of the cortical areas to stimulation of moderate duration lasting up to 15 s. Only one study assessed cortical activation during manual stimulation according to Vojta applied to an active site at the anterior thorax (Sanz-Esteban et al., 2018). However, methodological issues, such as unbalanced group sizes, a control site in a distant body part, and statistical maps uncorrected for multiple comparisons, do not permit drawing strong conclusions (Sanz-Esteban et al., 2018). To our knowledge, no previous imaging study evaluated immediate central effects of pressure stimulation of the foot according to reflex locomotion therapy (Vojta, 1973; Vojta and Peters, 2007), and in general, there are no fMRI data on responses to pressure foot stimulation delivered continuously over at least 30 s.

In summary, it is unknown whether the sensorimotor system response is influenced by a specific stimulation site, e.g., one used in reflex locomotion therapy. Furthermore, the link between the previously reported modulation of the motor task-evoked activation (Hok et al., 2017) and the stimulation-evoked responses remains to be established.

We hypothesized that, first, different body sites would differentially influence sensorimotor system during the stimulation, and second, that a site used in the reflex locomotion therapy would specifically activate the PMRF (Hok et al., 2017).

To address these hypotheses, we employed fMRI during block-designed sustained pressure stimulation at either an active (Vojta, 1973) or control site on the foot. We expected to identify the general activation pattern of cortical and subcortical areas involved in the central processing of sustained pressure stimulation of the foot while simulating clinical conditions of manual physiotherapy.

However, analysis of fMRI responses to sustained pressure stimulation has to address two physiological challenges: First, cortical response adapts rapidly within somatosensory areas, where it decreases exponentially over several seconds (Chung et al., 2015). Second, the activation of the presumed generators of the gradually developing widespread tonic motor reflex response would be expected to follow the same slow timecourse supposedly resulting from temporal summation over tens of seconds (Vojta, 1973). Both phenomena preclude the use of common models convolving a rectangular stimulus function with the canonical HRF. Therefore, we utilized a more flexible modeling approach, namely, a convolution with a set of FIR basis functions. The main hypotheses were tested quantitatively on a voxel-wise basis, evaluating within-subject differences between the active and control stimulation. Nevertheless, the FIR model does not assume any specific shape of the hemodynamic response, which may differ slightly among different brain areas and even within one functional system (Glover, 1999; Lewis et al., 2018). Since there is no common reference for the BOLD signal throughout the brain, interpretation of significant differences critically relies on identification of brain areas that significantly respond to the stimulation and the timecourse of these evoked responses. Therefore, on top of the paired analysis of stimulus-related differences, we have employed a correlation-based clustering approach to characterize the shape of group-wise BOLD responses at different levels of the sensorimotor system and to delineate subsystems that differentially respond to the stimulation and may have different functions.

## Materials and Methods

### Study Design

This proof-of-concept study has been conducted as a randomized cross-over experimental study in a single cohort of healthy adults to determine the central effects of the sustained manual pressure stimulation according to Vojta reflex locomotion (Vojta, 1973; Vojta and Peters, 2007) versus a sham stimulation.

### Participants

Thirty healthy volunteers enrolled in this study (16 females and 14 males, mean age 24.20, SD 1.92). The study participants were university students naïve to the technique of reflex locomotion, with no history of any neurological condition and no signs of motor disability. Twenty-seven subjects were right-handed and three were left-handed according to the Edinburgh handedness inventory (Oldfield, 1971). The study was carried out in accordance with World Medical Association Declaration of Helsinki. The study protocol was approved by the Ethics Committee of the University Hospital Olomouc and the Faculty of Medicine and Dentistry of Palacký University Olomouc under approval number 9.4.2013 and all participants gave their written informed consent prior to their inclusion in the study.

### Task and Procedures

Each fMRI session included 2 functional imaging acquisitions during 10 min of right foot stimulation. Prior to the stimulation, participants performed a sequential motor task with their right hand as described elsewhere (Hok et al., 2017). During the stimulation, participants were lying prone in the scanner bore with their eyes closed and were asked not to think about anything in particular. The stimulation was delivered in twelve blocks (each 30 s long) alternating with jittered rest to permit modeling of the extended hemodynamic response (Dale, 1999). In total, this resulted in 6 min of stimulation and 4 min of rest per acquisition run. The pressure was applied manually by an experienced therapist (MK or MŠ) using his/her thumb placed on one of two predefined sites located on the lateral side of the foot over bony structures and within the same dermatome (Foerster, 1933): either (1) the right lateral heel zone (heel stimulation, HS) according to Vojta (1973), or (2) a control site at the right lateral ankle (ankle stimulation, AS). The therapists were instructed to apply manual pressure similar to that routinely used during physiotherapy according to Vojta. The force applied was continuously recorded during the stimulation runs using a custom-made MRI-compatible calibrated pressure/force monitor (based on a FlexiForce sensor, Tekscan, South Boston, MA, United States). Throughout the acquisition, the stimulated limb was semi-flexed in the knee joint and supported above the scanner table by the therapist who maintained constant tactile contact with the participant’s foot to further simulate natural conditions of a therapeutic procedure. However, the use of a single stimulation site, the specific body position and stimulation duration, were chosen to elicit only partial motor response (Vojta and Peters, 2007), avoiding gross body movements and head displacement in the scanner bore.

After the session, participants reported discomfort/pain perceived during the stimulation using a VAS for discomfort/pain, with 0 (no discomfort/pain) and 10 (worst possible pain) marked as the extreme values. The discomfort/pain scores for HS and AS were compared using Wilcoxon two-sample signed rank test.

Every participant underwent two fMRI sessions, each involving either HS or AS. The session order was randomized and counter-balanced, and the participants were not informed in advance that the stimulation would be performed in one of two different sites. The sessions were scheduled at least 7 days apart (median interval was 70 days, range was 7–294 days).

### Data Acquisition

MRI data were acquired using 1.5-Tesla scanners (Siemens Avanto and Symphony, Erlangen Germany) with standard head coils. The scanning schedule was counter-balanced to account for any possible differences due to the scanner used. The subject’s head was immobilized with cushions to assure maximum comfort and minimize head motion. The MRI protocol included functional T_2_^*^-weighted BOLD images during task performance, acquired with gradient-echo EPI sequence (30 axial slices parallel to the anterior commissure-posterior commissure line, 5 mm thick, repetition time/echo time = 2500/41 ms, flip angle 70°, field of view = 220 mm, matrix 64 × 64) to provide 3.4 × 3.4 × 5.0 mm resolution. In total, 240 images were acquired per each functional run. Gradient-echo phase and magnitude field map images were acquired to allow correction of the echo planar imaging distortions. Anatomical high-resolution three-dimensional MPRAGE scan was acquired to provide the anatomical reference.

### Data Pre-processing

The fMRI data were processed using FEAT Version 6.00, part of FSL (FMRIB’s Software Library^1^), version 5.0.9 (Jenkinson et al., 2012). The FEAT pre-processing pipeline included: correction of B_0_ distortions using FUGUE (Jenkinson, 2003), motion correction using MCFLIRT (Jenkinson et al., 2002), non-brain removal using BET (Smith, 2002), and spatial smoothing using a Gaussian kernel with 8.0 mm FWHM. Functional data were registered to the individual’s anatomical reference image, which was subsequently normalized non-linearly to the MNI 152 standard space (Grabner et al., 2006). The fMRI data were then visually checked for susceptibility artifacts and two subjects were excluded due to an excessive signal loss in the brainstem. The final sample thus consisted of 28 subjects (16 females, 12 males, 25 right-handers).

Next, motion-related artifacts were removed from each time series using ICA-AROMA tool (Pruim et al., 2015a,b), followed by high-pass temporal filtering with sigma = 60.0 s. In a parallel preprocessing pipe-line, the ICA-AROMA noise components were removed from a dataset, which had no spatial smoothing applied. This dataset served for extraction of nuisance signal from six sources in the supratentorial white matter and one source in the lateral ventricles. The masks were based on the MNI 152 Harvard-Oxford cortical atlas labels at 95 and 85% probabilistic threshold, respectively (Desikan et al., 2006). The white matter mask was split along the orthogonal planes into 6 areas roughly corresponding to the frontal (*Y* ≥ 0 mm), parietal (0 mm > *Y* ≥ −36 mm, *Z* ≥ 18 mm) and occipital white matter (*Y* < −36 mm), excluding the deep white matter around basal ganglia. From each source, the first eigenvariate was used to represent the non-neuronal signal.

### Statistical Analysis of Imaging Data

The statistical analysis of the time-series was carried out in all remaining 28 subjects using FILM with local autocorrelation correction (Woolrich et al., 2001). To account for habituation with minimum assumptions, the onsets of stimulation blocks were convolved using a set of FIR basis functions instead of the canonical HRF. The GLM thus consisted of 9 delta functions (i.e., 9 temporally shifted unit spikes approximating Dirac delta function) that covered a 45 s time window (30 s on task and 15 s off task) aligned with the onset of each block with a 5 s (2 repetition times) steps to avoid noise over-fitting (Liu et al., 2017). To suppress residual physiological noise, the final model included also 6 nuisance signal regressors from the white matter and 1 from the ventricles.

The resulting beta parameters (in FSL terms, contrasts of parameter estimates or COPE) were carried over to a middle-level analysis in order to account for repeated measures in each subject. At this step, each time point (i.e., basis function) was still considered independent and analyzed separately for each subject. Since only within-subject effects were modeled at this point, the middle-level analysis was carried out using the fixed effects mode in FEAT. To test the main hypotheses, three within-subject models were designed and evaluated in parallel pipelines: In the first one, the beta parameters from each session (involving either HS or AS) were averaged separately, resulting in Contrasts 1 (HS) and 2 (AS). These contrasts represent the mean condition effects related either to HS or AS. In the second model, the functional series from both sessions were pooled together, providing Contrast 3 (HS + AS). This contrast was necessary to obtain a mean activation map for HS and AS, which would provide common clusters for a *post hoc* ROI analysis. Finally, the within-subject differences were assessed on a voxel-wise basis by subtracting the beta parameters from both sessions, yielding Contrast 4 (HS − AS).

**FIGURE 1:**
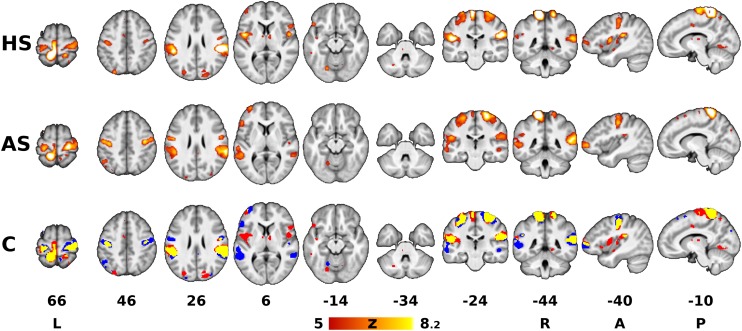
Areas associated with sustained pressure stimulation. The red-yellow Z statistical overlays in the top and middle rows represent significant *F*-tests of mean response to heel stimulation (HS) and ankle stimulation (AS). The bottom row shows the binary conjunction (C) of HS and AS (red = heel, blue = ankle, yellow = conjunction of both). The images were superimposed on top of a gray-scale mean T_1_-weighted background image. Clusters of activation were determined by *Z* > 5 and thresholded at corrected *p* < 0.05. The slices are numbered according to coordinates in the Montreal Neurological Institute (MNI) 152 standard space template. The right is right, according to neurological convention.

**FIGURE 2:**
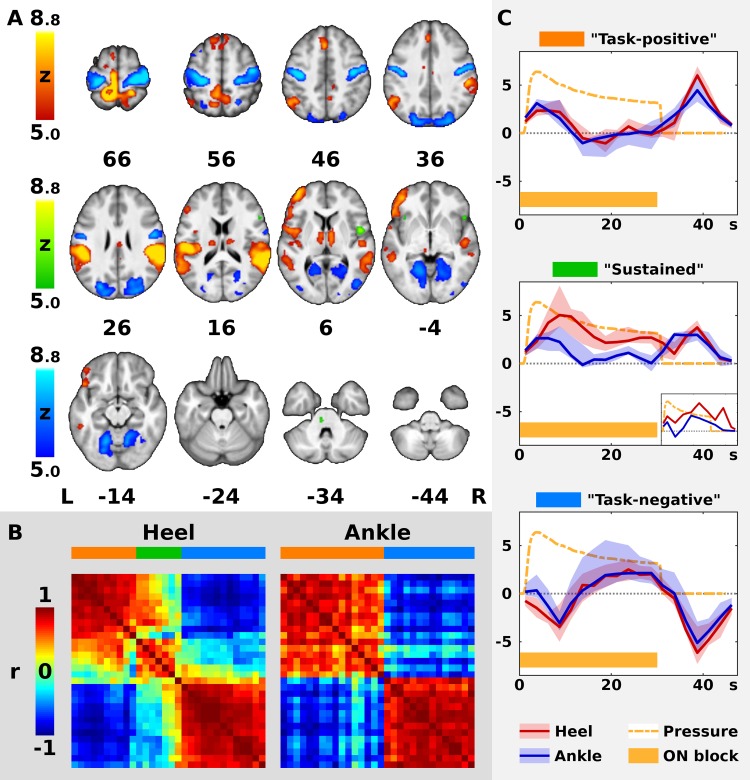
Timecourses of BOLD signal in the significant clusters. In panel **(A)**, the color Z statistical overlays represent together significant *F*-test of mean pooled response to both heel (HS) and ankle stimulation (AS). Significant clusters were separated into three color-coded groups (red, green, and blue) according to the shape of hemodynamic response function (HRF), as explained in panels **(B,C)**. For remaining conventions in panel **(A)**, see Figure 1. In panel **(B)**, the left matrix (Heel) represents cross-correlations of hemodynamic responses in 30 largest clusters from panel **(A)** as measured during HS, whereas the right matrix (Ankle) represents cross-correlations observed during AS. Both matrices are identically ordered according to the minimal Euclidean distance between neighboring clusters in Heel condition (see Methods). Note the two well-formed anti-correlated subsystems in Ankle condition (right matrix), encoded in red and blue on the horizontal bar above the matrix. In Heel condition, another subsystem emerges in addition to the previous two. The three networks are encoded in red, green and blue. In panel **(C)**, the plots display median (solid dark line) and inter-quartile range (semi-transparent fill) of HRF across all clusters in each network from panels **(A,B)** (from top to bottom: red, green, and blue). In the middle plot, a smaller plot represents a single cluster with a distinct timecourse during AS. Abscissa represents time since the block onset in s, whereas ordinate represents fitted blood oxygenation level-dependent response in arbitrary units. Dashed orange line shows the average applied pressure function (scaled to fit the plot), whereas the orange bar below indicates the duration of the stimulation block (ON).

**FIGURE 3:**
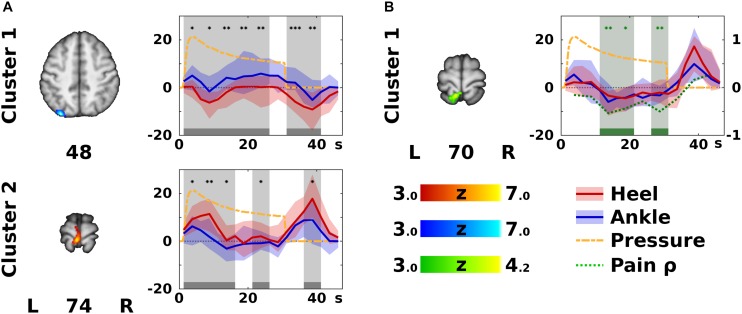
Significant differences according to stimulation site. In panel **(A)**, the color Z statistical overlays represent significant *F*-test of within-subject differences between the heel (HS) and ankle stimulation (AS), i.e., Contrast 4 (HS – AS). The two clusters (labeled anatomically in Table 1) are coded either in red, if HS yielded higher activation than AS, or in blue, if the opposite was the case. The plots on the right side of each slice display median (solid dark line) and inter-quartile range (semi-transparent fill) of the modeled hemodynamic response function in the specified cluster across all subjects (HS in red, and AS in blue). Gray bars and background indicate epochs (each epoch represents one finite impulse response basis function) that significantly differed between HS and AS (Wilcoxon signed rank test). Differences were significant at uncorrected ^*^p < 0.05, ^∗∗^*p* < 0.01, or ^∗∗∗^*p* < 0.001 (*post hoc* confirmatory analysis). For remaining conventions see Figure 2. In panel **(B)**, a cluster showing significant correlation between pain difference HS – AS and activation difference (HS – AS) is displayed in green [see Contrast 5 (Pain) in the Methods]. In the corresponding timecourse plot, green bars and background indicate significant correlation according to Spearman’s correlation coefficient (ρ), which is plotted as a green dotted line (the ordinate range is marked on the right). Note that correlations were significant in different areas and epochs than the significant differences between activation levels in HS and AS. For remaining conventions see panel **(A)**.

In the final third-level analysis, group-wise effects for all within-subject contrasts were evaluated. The group model consisted of one regressor for each basis function and an *F*-test collapsing all 9 basis functions to assess the overall effect over the entire stimulation block. In Contrast 4 (HS − AS), additional linear covariates were included to account for the time difference between the two sessions and for individual differences in self-rated discomfort/pain intensity (condition H – condition A), with an additional *F*-test to evaluate the average discomfort/pain effect [Contrast 5 (Pain)]. The random effects analysis was performed using FLAME (FMRIB’s Local Analysis of Mixed Effects) stage 1 (Woolrich et al., 2004). The whole-brain analysis was limited to the MNI standard brain mask (Grabner et al., 2006) minus a white-matter mask derived from the Harvard-Oxford probabilistic atlas (Desikan et al., 2006) using a conservative probability threshold of 95% as defined in the Section “Data Pre-processing.” The masked Z (Gaussianised T) statistic images were thresholded using clusters determined by *Z* > 5 in case of Contrasts 1 to 3 (Figures 1, 2), or *Z* > 3 in case of Contrasts 4 and 5 (Figure 3). The FWE corrected cluster significance threshold was *p* < 0.05 (Worsley, 2001). Clusters in the thresholded maps were objectively labeled using the Harvard-Oxford Cortical and Subcortical Structural Atlases (Desikan et al., 2006), and the Probabilistic Cerebellar Atlas (Diedrichsen et al., 2011). Cytoarchitectonic labels were derived from the Jülich Histological Atlas (Eickhoff et al., 2007). The resulting statistical images were rendered in Mango v4.0 (Research Imaging Institute, UT Health Science Center at San Antonio, TX, United States^2^).

### *Post hoc* ROI Analysis – Mean Condition Effects

To assess temporal features of the hemodynamic responses in the areas significantly activated or deactivated by either stimulation, a *post hoc* ROI analysis was performed and visualized using custom scripts created in Matlab version R2017b and the Statistics Toolbox (MathWorks, Natick, MA, United States). Only clusters in Contrast 3 (HS + AS) containing more than 5 voxels were considered.

First, average group-wise activations were investigated. Using the cluster mask from Contrast 3 (HS + AS), group-wise beta parameters were extracted from the Contrasts 1 (HS) and 2 (AS) for each time point (i.e., basis function). The representative cluster-wise values were obtained using median of beta parameters in each cluster. Vectors of 9 consecutive median beta parameters in each cluster thus provided cluster-wise timecourses, each representing median response during a single stimulation block and the subsequent rest.

To assure that the extracted medians represented a homogeneous population of voxels, each median timecourse was correlated using Pearson’s correlation coefficient with the first PC obtained from the same cluster using singular value decomposition (Wall et al., 2003, 91–109). In case of low correlation between the median of the whole cluster and the first PC (*r* < 0.7), the median was extracted only from a subset of voxels highly correlated with the first PC in both HS and AS (*r* > 0.75).

The resulting representative cluster-wise timecourses (i.e., vectors of the median beta parameters) were then correlated with each other using Pearson’s correlation coefficient, providing one correlation matrix for HS and one for AS. Next, hierarchical clustering was applied to both correlation matrices in order to distinguish “subsystems” (sets of clusters) with similar hemodynamic responses. Agglomerative clustering trees were built using unweighted average distance algorithm and Euclidean distance as a dissimilarity measure (Rencher and Christensen, 2012). The optimal number of resulting subsystems was indicated using Caliński-Harabasz criterion (Caliński and Harabasz, 1974).

For visual comparison, the correlation matrix for AS was re-ordered according to the correlation matrix for HS (Figure 2B). Finally, the original HRF in each cluster was reconstructed by multiplying the convolution matrix and the group-wise beta weights of each FIR regressor (Figure 2C).

### *Post hoc* ROI Analysis – Within-Subject Differences

Further *post hoc* analysis was performed to determine the timing and directionality of differences detected in Contrast 4 (HS − AS). This was done by extracting the median within-subject beta parameters from Contrasts 1 (HS) and 2 (AS) within the boundaries of the clusters from Contrast 4 (HS − AS). To identify time points of significant differences, corresponding beta parameters for HS and AS were compared using paired Wilcoxon signed rank test at *p* < 0.05 (*post hoc* confirmatory analysis without additional correction). Finally, the differences in activation levels in clusters from Contrast 5 (Pain) were correlated with discomfort/pain rating difference using Spearman’s correlation coefficient and marked significant at *p* < 0.05. These results are presented in Figure 3.

## Results

### Behavioral Data

In all subjects, the therapist observed discrete irregular focal muscle contractions in the stimulated extremity during stimulation, but no gross limb or trunk movements.

For technical reasons, continuous pressure recordings were only obtained in 15 subjects. The mean force applied at the sensor during HS was 22.33 N (SD = 11.64 N) and 26.45 N (SD = 9.72 N) during AS. The difference was not significant (*p* = 0.32, two-sample *t*-test). A paired *t*-test was possible in 11 subjects with a non-significant difference (*p* = 0.22, mean difference HS − AS = −3.94 N, SD = 9.96 N).

After HS, the median reported discomfort/pain intensity (VAS) was 1.85 (range 0–6.9), while it was 0.90 after AS (range 0–5.5). HS was thus associated with significantly higher discomfort/pain intensity than AS (*p* < 0.01, Wilcoxon signed rank test), with median difference 1.25 (range −5.0–6.4). The difference in discomfort/pain rating has been therefore included as a covariate in the Contrast 4 (HS − AS).

### Imaging Results

#### Spatial Maps of Mean Condition Effects

Group Contrasts 1 (HS) and 2 (AS) yielded separate Z statistical maps depicting areas with significant response either to HS or to AS (Figure 1). The areas involved in the somatosensory processing of the pressure stimulation of each site overlapped partially (spatial correlation between thresholded Z statistical maps for HS and AS was 0.56 using Pearson correlation coefficient). The overlapping areas (binary conjunction, see yellow overlay in Figure 1, row C) included mainly the left dorsomedial primary somatosensory and motor cortex (S1 and M1, respectively) in the somatotopic representation of the stimulated lower limb and the bilateral parietal operculum cortices (secondary somatosensory cortex, or S2). Less extensive overlap was observed in the more posterior right postcentral gyrus and SPL, i.e., ipsilateral to the stimulated limb. Both stimulation sites were also associated with signal changes in bilateral dorsolateral sensorimotor cortex (SMC, i.e., S1 and M1) in the somatotopic representation of the upper limb and face (Long et al., 2014). These were later identified as transient deactivations, see below. Further similarities between the responses to stimulation at either site were found in the left prefrontal and bilateral parieto-occipital cortices, bilateral lingual gyri and thalami, but the involved areas mostly did not overlap. Several qualitative differences were observed: AS was associated with more involvement of temporal and prefrontal areas in the left hemisphere, whereas HS elicited responses in the left insular and bilateral frontal operculum cortices and the brainstem in the contralateral (left) pons.

The analysis of pooled data (Contrast 3 [HS + AS], sum of all color overlays in Figure 2A) yielded significant effects in all areas associated with either HS or AS alone. Therefore, a complete list of clusters with anatomical labels is only provided for Contrast 3 (HS + AS; see Supplementary Table S1).

#### *Post hoc* ROI Analysis

The ROI analysis of the clusters obtained from Contrast 3 (HS + AS) was limited to the 30 biggest clusters with more than 5 voxels (see Supplementary Table S1 for a complete list). The median group-wise beta parameters were highly correlated with the first principal component in all but one cluster, namely, Cluster 1. In this cluster, the first PC was dominant for both stimulation sites (*r* > 0.75) in 2,798 voxels (47.5% of the original cluster size), which were used to extract representative response time-course. The remaining voxels were not considered.

In the 30 evaluated clusters, the modeled BOLD responses could be mostly separated into two distinct subsystems with anti-correlated timecourses (Figure 2B). This was especially apparent in AS. Therefore, all clusters in AS condition and most clusters in HS condition were labeled either as “task-positive” or “task-negative” based on the sign of the immediate BOLD signal change. According to the timecourse plots, the median activation in the task-positive subsystem (“Task-positive” plot in Figure 2C) increased immediately after the stimulation onset and peaked at 3.75 s, namely, at the center of the second volume after onset. It decreased back to baseline as early as 10 s after onset. Following the stimulation offset, activation transiently increased again and remained positive 0 to 17.5 s after offset, peaking at 8.75 s. As opposed to the task-positive areas, the responses in the second subsystem (“Task-negative” plot in Figure 2C) involved deactivations at the onset and at the offset of the stimulation. The median response remained negative 5 to 12.5 s after onset and 5 to 17.5 s after offset. Please note that the real time resolution of the plots is roughly 5 s, which is the approximate width of a single regressor spanning 2 TRs.

Whereas there were only two subsystems with homogeneous responses in AS, a third type of response could be distinguished in HS (see dendrograms in Supplementary Figure S1). The 23 clusters with consistent task-positive or task-negative responses, which were similar in both conditions are represented by red and blue overlay, respectively, in Figure 2A. The responses in the remaining 7 clusters in HS condition followed a distinct timecourse that deviated from the common task-positive or task-negative pattern (compare the matrices in Figure 2B; see also Supplementary Figure S1, dendrogram “Heel”). Six out of these clusters were task-positive in AS and one was task-negative in AS, including the right frontal and central opercular cortex, inferior frontal gyrus, frontal orbital cortex, bilateral anterior insular cortex, left paracingulate gyrus and the left pons (see green overlay in Figure 2A). In these clusters, the initial response in HS condition remained positive for the duration of the stimulation block (peak at 8.75 s after onset) instead of dropping immediately to baseline. After the offset, the second positive response could be observed at 8.75 s after offset. Therefore, the subsystem was labeled as “sustained task-positive” (compare the red solid line representing HS to the blue line representing AS in “Sustained” plot in Figure 2C).

#### Within-Subject Differences Between Conditions

Contrast 4 (HS − AS) yielded a map of average within-subject differences between HS and AS (Figure 3A), as well as the interaction with the self-reported discomfort/pain intensity (Figure 3B). The differences between HS and AS were observed in the IPL (area PGp; Cluster 1 in Figure 3A) and in the left primary motor (M1) and PMC in the somatotopic representation of the lower limb (BA 4a and 6; Cluster 2 in Figure 3A). The discomfort/pain effect [Contrast 5 (Pain)] was observed in the left SPL (BA 7A and 5L; Cluster 1 in Figure 3B) posterior to the Cluster 2 in Contrast 4 (HS − AS). A complete list of clusters with their anatomical labels is provided in Table 1.

**TABLE 1:** List of clusters of significant differences according to stimulation site.

**Contrast**	**Cluster index**	**Anatomical atlas labels**	**Cytoarchitectonic atlas labels**	**Volume (cm^3^)**	**Cluster *p***	**Z_*max*_**	**Z_*max*_ MNI coordinates [x,y,z (mm)]**
Contrast 4: HS – AS	1	100.0% L Lateral Occipital C, s. d.	81.5% L Inferior Parietal Lobule PGp 7.1% L Inferior Parietal Lobule PGa 5.1% L Superior Parietal Lobule 7A	2.81	0.003	7.00	−30, −80, 48
	2	48.5% L Postcentral G 36.5% L Precentral G 8.8% R Precentral G 6.2% R Postcentral G	65.0% L Primary Motor C BA4a 18.1% L Premotor C BA6 8.5% R Primary Motor C BA4a	2.08	0.014	6.74	−4, −36, 74
Contrast 5: Pain effect	1	53.0% L Superior Parietal Lobule 29.8% L Postcentral G 12.1% L Lateral Occipital C, s. d. 5.1% L Precuneous Cortex	46.0% L Superior Parietal Lobule 5L 45.5% L Superior Parietal Lobule 7A	1.58	0.043	4.16	−8, −48, 70

*Table lists significant *F*-test clusters in Contrast 4 (HS – AS), i.e., the differences between heel and ankle stimulation, and in Contrast 5 (Pain), i.e., the pain effect. Anatomical and cytoarchitectonic labels are provided including the proportion of labeled voxels. Only labels consisting at least 5% of activated voxels are shown. Note that cerebellar labels may overlap with cortical labels and that cytoarchitectonic labels do not cover the whole brain. Abbreviations: C, cortex; BA, Brodmann area; G, gyrus; MNI, Montréal Neurological Institute; R, right; s. d., superior division; Zmax, maximum Z score.*

The ROI analysis of clusters in Contrast 4 (HS − AS; see Table 1) revealed that the modeled BOLD response in the left M1 and PMC (Cluster 2 in Figure 3A) was significantly higher in HS condition. This was observed mostly during short activation increases after stimulation onset and offset. In contrast, activation levels in the left IPL (Cluster 1 in Figure 3A) were higher in AS condition than in HS condition. The differences in the IPL were spread almost over the entire stimulation block and the subsequent rest.

The ROI analysis of the cluster obtained from Contrast 5 (Pain) showed that the discomfort/pain difference (HS − AS) was negatively correlated with the difference in activation levels (HS − AS). The significant correlations were detected during the sustained phase of the stimulation (Figure 3B).

## Discussion

In this section, we discuss the main findings in the following order: brain structures associated with the pressure stimulation of the foot, the dynamics of the BOLD responses, deactivations observed during the stimulation, and the site-specific differences, which are the main novel findings of this study.

### Patterns of Activation Associated With Pressure Stimulation

Using a FIR model to deconvolve the hemodynamic response, we have confirmed that sustained peripheral pressure stimulation influences multiple elements of the sensorimotor system. The stimulus-related activation increases that we observed mainly in the contralateral S1 and bilateral S2 regardless of stimulation site (Figure 1) are consistent with previous descriptions of the core somatosensory network activated during pressure stimulation applied either at the upper or the lower limb (Hao et al., 2013; Miura et al., 2013; Chung et al., 2015). Further consistent activations that we detected in the contralateral dorsomedial M1/PMC have only been observed in lower limb stimulation (Hao et al., 2013; Miura et al., 2013), whereas activations in the ipsilateral dorsomedial S1/SPL have been previously reported only in one study (Miura et al., 2013). Other brain structures activated either by HS or AS, or observed in the pooled analysis [Contrast 3 (HS + AS)], such as frontal, insular or cingulate cortices and bilateral thalami, also agree with previous studies (Miura et al., 2013; Chung et al., 2015). Therefore, the described general activation pattern during sustained pressure stimulation of the foot may be considered rather independent of stimulation site and duration.

### Temporal Features of the BOLD Responses

Apart from the localization of signal changes, we also deconvolved the timecourse of the regional hemodynamic responses to natural manual pressure stimulation.

First, this allowed us to confirm that fast adaptation (Chung et al., 2015) occurs also during longer and repeated sustained stimulation. The sensation of static mechanical pressure is believed to be conducted via slowly adapting I (SA-I) afferents (Johansson and Flanagan, 2009). These afferents adapt exponentially to static stimuli (indentation or vibration) with a time constant of 8.4 s (Leung et al., 2005). Considering the time lag of the BOLD response, the activations in our data in the task-positive areas (coded in red in Figures 2A,C) occurred and diminished within the expected time window (0 to 10 s after onset), which is in overall agreement with previous observations (Chung et al., 2015).

Second, we show that an equal response follows the release of pressure (Figure 2C). Similar response has been observed after offset of sustained non-nociceptive vibratory (Marxen et al., 2012) or electrical stimulation (Hu et al., 2015), but it has not been reported so far in sustained pressure stimulation (Chung et al., 2015). Importantly, the offset responses have been shown to occur only after non-nociceptive stimulation (Hu et al., 2015), suggesting that the task-positive areas with offset responses in our data (red overlay in Figure 2A) were not associated with processing of painful sensations and could potentially receive input mediated by rapidly adapting (RA) afferents (Hu et al., 2015), but this has to be confirmed by future electrophysiological studies.

Regarding the magnitude of the offset responses, it should be noted that both positive and negative offset responses were apparently of higher amplitude and longer duration (0 to 17.5 s after offset) than the responses at the stimulation onset. We speculate that the reason might be to some extent related to our experimental design: the offset pressure decrease may have been on average more abrupt and less variable than the pressure increase at the block onset. As a result, onset responses might by slightly “blurred” in time.

### Deactivations Associated With Pressure Stimulation

In addition to areas activated during the stimulation, we also report a complementary set of brain areas, which were transiently suppressed by the stimulation and the pressure release. Similar inhibition in the bilateral S1 and M1 has been previously documented during vibrotactile finger or tactile foot stimulation (Hlushchuk and Hari, 2006; Tal et al., 2017). We extend this observation by showing that such suppression occurs also in response to sustained pressure stimulation of the lower limb. In line with Tal et al. (2017), we show that foot stimulation deactivates sensorimotor cortices in the bilateral somatotopic representations for upper limbs and face (blue overlay in Figure 2A) as defined by Long et al. (2014). A new finding in the context of lower limb stimulation is the deactivation in areas outside the sensorimotor system, such as the temporal and occipital cortices. Similar cross-modal deactivations have been observed in humans only during somatosensory processing of tactile input from the upper limbs and they have been speculated to enhance the somatosensory processing by suppressing unnecessary sensory input (Kawashima et al., 1995; Merabet et al., 2007; Ide et al., 2016).

The observed deactivations are unlikely to be caused by local redistribution of the blood flow (hemodynamic steal) as most of the areas showing differential responses are supplied by different main cerebral arteries (Tal et al., 2017). Electrophysiological evidence from direct intracortical recordings suggests that negative BOLD response is associated with suppressed neuronal activity in the deep cortical layers (Boorman et al., 2010; Yin et al., 2011). Simultaneous fMRI/EEG recordings in humans show considerable correlation between the EEG mu power and BOLD signal decrease, confirming its neuronal origin (Mullinger et al., 2014). Recent data show that inhibitory neurons may also contribute to the positive hemodynamic response, hence, deactivations could conversely reflect decreased neuronal activity of both excitatory and inhibitory cells (Vazquez et al., 2018). However, there is also evidence suggesting that the deactivated areas are not necessarily always “shut down.” Decrease in BOLD signal and cerebral blood flow may be at least in some cases accompanied by increased spiking (Hu and Huang, 2015) and/or glucose uptake (Devor et al., 2008). Since the underlying neuronal processes and functional role of negative hemodynamic responses are not yet clearly understood, they should be interpreted with caution (Tal et al., 2017).

### Differences Between the Heel and Ankle Stimulation

#### Voxel-Wise Within-Subject Comparison

Compared to control stimulation, HS was associated with significantly increased activation in the left M1/PMC (somatotopically lower limb area; see Cluster 2 in Figure 3A) and decreased activation in the left IPL.

Activations in the contralateral motor cortex have already been observed during pressure stimulation of the lower limb (Hao et al., 2013; Miura et al., 2013) as discussed in the Section “Patterns of Activation Associated With Pressure Stimulation.” Although both AS and HS were associated with transient activations in the M1 representation for the stimulated limb, the results indicate higher neuronal activity during HS (Figure 3A, Cluster 2). This may have several possible reasons: A shift in somatosensory representation is unlikely as the activations in the postcentral gyrus did not differ. While the local stimulation site properties may also influence the activations, we believe that there were no sources of bias other than those, which may be in fact important for the reflex locomotion therapy (see also Limitations). The increased motor activation may also be a secondary phenomenon, for instance, reflecting pain-evoked movements (Apkarian et al., 2005). Since the Contrast 4 (HS − AS) was controlled for the difference in discomfort/pain rating, we consider the M1/PMC activation differences to be less likely pain-related (see also Limitations). Next, the observed difference in the M1/PMC may result from an incipient involuntary muscle response to stimulation according to Vojta and may be mediated by a different, possibly subcortical or brainstem structure (Vojta, 1973; Laufens et al., 1991; Hok et al., 2017). Finally, the increased motor activation during HS may also represent a site-specific difference in sensorimotor integration. It remains unknown at which level the sensory input is redirected to the motor cortex. It may either reflect a direct interaction between the adjacent somatosensory and motor cortices (Kaelin-Lang et al., 2002), or a parallel bottom-up thalamo-cortical pathway (Huffman and Krubitzer, 2001) or collaterals of the spinothalamic pathway (Kayalioglu, 2009). Such direct influence of sensory input on motor cortex function is supported by electrophysiological evidence using sustained electrical (Golaszewski et al., 2012), vibratory (Marconi et al., 2008), or vibrotactile (Christova et al., 2011) stimulation, which shows outlasting effects on motor cortex excitability, possibly by affecting inhibitory GABA-ergic intracortical circuits (Ziemann et al., 1996).

In contrast to the task-positive motor activations, the differences in the IPL (Figure 3A, Cluster 1) are more likely related to cross-modal deactivations (Kawashima et al., 1995; Merabet et al., 2007; Ide et al., 2016) as discussed in the Section “Deactivations Associated With Pressure Stimulation.” The posterior IPL (cytoarchitectonically the area PGp) is considered a part of the default mode network, specifically its medial temporal lobe subsystem (Igelström and Graziano, 2017). Similar stimulus-related deactivations in parts of the default mode network have been previously observed during sustained electrical stimulation (Hu et al., 2015). These deactivations varied over different phases of stimulation, left IPL being predominantly deactivated during the onset phase of periodic stimuli (Hu et al., 2015). Nevertheless, the role of those deactivations remains unclear. Since cognitive processes were not explicitly controlled in this study, we can only speculate that the higher amplitude of deactivations in the IPL-PGp could mean that the sensory input associated with HS was suppressing internally driven cognitive processes, possibly by drawing more externally oriented attention.

#### Comparison of Group-Wise Activation Patterns

During HS, average activation in several areas followed a timecourse with more sustained positive BOLD response (red solid line in “Sustained” plot in Figure 2C), whereas in AS, only transient onset/offset activations were detected (blue solid lines in Figure 2C). Some of these areas, including insular cortices and pons, were not observed in the group-wise map for AS condition, but they were detected in HS (Figure 1).

The involvement of the insular cortex in HS may in fact reflect increased discomfort/pain ratings during HS since the insular cortex is known to participate in emotional processing of pain (Apkarian et al., 2005; Kurth et al., 2010; Hu et al., 2015). However, other explanations remain possible as there was no significant correlation with discomfort/pain intensity difference in the insulo-opercular areas in Contrast 5 (Pain). For instance, anterior insula also significantly contributes to the control of autonomic responses (Beissner et al., 2013) and various cognitive and affective processes (Kurth et al., 2010; Uddin, 2015). Indeed, stimulation according to Vojta has been associated with various autonomic responses (Vojta and Peters, 2007), but our parallel investigation of cardiac autonomic responses in a similar cohort of healthy subjects did not indicate any site-specific effect of HS which would interfere with our current results (Opavský et al., 2018).

Another structure associated with HS (but not significantly with AS, see Figure 1, row C) was included in the sustained task-positive subsystem (green overlay in Figure 2A) and located in the pontine tegmentum. The area most likely encompasses the PRF and pontine nuclei (Nieuwenhuys et al., 2008). These are adjacent to the PMRF in which we have previously observed modulation of motor-related activation after sustained pressure stimulation (Hok et al., 2017). Based on that observation, we have previously speculated that the PMRF might play a role in the therapeutic stimulation according to Vojta (Hok et al., 2017). While the current study does not provide further direct evidence for the specific role of PRF or PMRF in the physioterapeutic effects of pressure stimulation, the sustained activation in the PRF during HS (see “Sustained” plot in Figure 2C) provides a ground for potential interaction between the PRF and the more caudal PMRF.

In humans, the brainstem reticular formation, and more specifically the PMRF, is suggested to exert anticipatory postural control before gait initiation (Takakusaki, 2013). It also activates during the imagery of standing (Jahn et al., 2008) and walking (la Fougère et al., 2010). Most importantly, however, stimulation of the PMRF elicits bilateral asymmetrical motor patterns in cats (Dyson et al., 2014) and monkeys (Hirschauer and Buford, 2015), which can be related to stereotypic tonic responses observed by Vojta (1973) and Vojta and Peters (2007).

### Implications for Physiotherapeutic Techniques

Our findings indicate that sustained pressure stimulation affects the sensorimotor system on a global scale. While some areas (e.g., the primary SMC for the foot) respond with increased activation, other regions (such as the primary SMC for the hand and face) became transiently suppressed. This effect seems to be non-specific and independent of the stimulated site. However, specific effects during the HS were observed as well.

Pressure stimulation is an integral part of number of physiotherapeutic techniques, such as reflex locomotion (Vojta and Peters, 2007), clinical massage, acupressure (Wong et al., 2016), reflexology, or myofascial trigger point therapy (Smith et al., 2018). Whereas in reflex locomotion, the choice of exact stimulation site is pre-defined (Vojta and Peters, 2007), other techniques, such as myofascial trigger point therapy, do not rely on specific body site (Smith et al., 2018). Our data show that even non-specific pressure stimulation may evoke far-reaching effects throughout the brain, including the motor system, which is relevant for physiotherapy. Whether the observed cortical activations/deactivations in the current study have any outlasting and clinically significant impact, cannot be established without further studies with comprehensive protocols employing imaging and repeated behavioral testing.

Our choice of the specific stimulation site was motivated by the stimulation according to Vojta, which is known to induce significant modulatory motor after-effects, e.g., facilitation of voluntary movements that outlast the stimulation (Laufens et al., 1995). Our current data provide further evidence that sustained pressure stimulation may influence multiple sensorimotor areas (including representations of distant extremities) without any evoked gross motor activity. The site-specific effects were local, i.e., confined to the motor cortex adjacent to the primary somatosensory representation of the stimulated limb. While the co-activation in the primary motor and premotor cortex of the stimulated (lower) limb seems to be relatively non-specific (Hao et al., 2013; Miura et al., 2013), we show that it can be augmented by stimulation at certain sites, such as the lateral heel zone according to Vojta (1973).

However, the fact that we deliberately did not elicit any consistent gross involuntary motor responses limits our ability to connect our observations with the anatomical structures responsible for the control of the motor patterns observed during the reflex locomotion therapy (Vojta, 1973). Still, we expand our recent observation of the modulatory motor after-effects in the PMRF (Hok et al., 2017) by showing that HS is associated with sustained activation in the nearby PRF, which was not observed during control stimulation. We speculate that an interaction (possibly top-down) between these brainstem nuclei might be responsible for the global motor effects of the reflex locomotion therapy.

The need for targeted stimulation of empirically chosen sites in reflex locomotion resembles other therapeutic techniques, such as acupuncture. In (electro)acupuncture, a considerable number of fMRI studies compared brain activations in response to the “active” and sham sites, but results are often conflicting (Qiu et al., 2016). A specific activation increase in response to lower limb stimulation was observed in the contralateral primary motor cortex (Wu et al., 2002; Usichenko et al., 2015) in agreement with our results, suggesting that there might be a more universal mode of action common for both reflex locomotion and acupuncture. However, differences in many other brain areas not corresponding to our results, including frontal and temporal cortices and limbic structures, were also observed (Wu et al., 2002; Usichenko et al., 2015), therefore, other mechanisms might be involved as well. A head to head comparison would be required to assess this.

### Limitations

Because of the whole-brain fMRI acquisition, the spatial resolution of the T_2_^*^-weighted MR images may limit assignment of activation foci to a single anatomical area in a small structure such as the brainstem. Nevertheless, functional MR imaging of the brainstem was successfully performed in the past using spatial resolution and hardware comparable to ours (Jahn et al., 2008). Moreover, data acquisition using a 1.5-T scanner may be less prone to magnetic susceptibility artifacts that affect higher-field 3-T scanners more severely, despite their superior signal to noise ratio.

Furthermore, the observed activation differences between HS and AS might be to some extent influenced by concomitant discomfort/pain. In this study, the HS was indeed rated more unpleasant/painful than the AS. This is in line with the reports that therapeutic stimulation according to Vojta is associated with concomitant pain (Müller, 1974). While electromyographic recordings from the stimulated and non-stimulated limbs would be needed in future studies to completely exclude the possibility of pain-related movements, the overall discomfort/pain intensity ratings in this study were quite low in both conditions (median VAS in HS 1.9, in AS 0.9). In the whole-brain analysis, the differences between HS and AS were controlled for the discomfort/pain effect. In fact, the interaction between discomfort/pain (self-rated discomfort/pain intensity difference) and stimulation modality (HS or AS) was observed in different areas than the differences between stimulation modalities alone. The posterior parietal areas have been previously reported as parts of the pain perception network (Apkarian et al., 2005).

Further potential bias may arise from differences in local characteristics between the two stimulation sites, such as density of sensory nerve endings, soft tissue properties or bony structures below the skin. As mentioned in Methods, both sites were within the same dermatome (Foerster, 1933). Since the active site (heel) was defined by Vojta (1973), the control site was carefully chosen to match as many properties as possible, i.e., neither site was located at the foot sole, but rather on the lateral aspect of the foot. We do not consider either site to contribute specifically to any motor or balance control function. Conversely, it is likely that some of the local site properties indeed play a role in the therapeutic effect of the reflex locomotion therapy, but further studies testing multiple sites in different dermatomes over different types of tissues would be needed to elucidate this.

## Conclusion

We have confirmed that sustained manual pressure stimulation of the foot is associated with extensive activation throughout the sensorimotor system and, for the first time in the context of the pressure stimulation, that it is accompanied by equally prominent cross-modal deactivations, including the occipital cortices and sensorimotor representation of the upper limbs and face. The timecourse data confirm fast adaptation of the sensory processing system, but also reveal previously underreported transient responses related to the stimulation offset. We further report that sustained pressure stimulation of the (active) site at the heel, which is used in the reflex locomotion therapy, elicited increased cortical activation in the primary motor representation of the stimulated limb and decreased activation in the posterior parietal cortex. Moreover, the stimulation of the active site was associated with a more sustained BOLD response in the insulo-opercular cortices and contralateral pons. We suggest that the increased motor activation and involvement of the pontine reticular formation could be associated with the previously observed motor after-effects of reflex locomotion therapy.

## Data Availability

The raw data supporting the conclusions of this manuscript will be made available by the authors, without undue reservation, to any qualified researcher.

## Ethics Statement

This study was carried out in accordance with the recommendations of Ethics Committee of the University Hospital Olomouc and the Faculty of Medicine and Dentistry of Palacký University Olomouc with written informed consent from all subjects. All subjects gave written informed consent in accordance with the Declaration of Helsinki. The protocol was approved by the Ethics Committee of the University Hospital Olomouc and the Faculty of Medicine and Dentistry of Palacký University Olomouc.

## Author Contributions

PHo has substantially contributed to the design of the work, data acquisition, analysis and interpretation, and drafted the manuscript. JO has substantially contributed to the conception, design, and interpretation of the work, and substantially revised the manuscript. RL has substantially contributed to the data analysis and drafted parts of the manuscript. MK has substantially contributed to the design of the work and data acquisition. MŠ has substantially contributed to the data acquisition. ZT has substantially contributed to the data acquisition and substantially revised the manuscript. PK has substantially contributed to the conception of the work. PHl has substantially contributed to the conception and design of the work, data acquisition, and substantially revised the manuscript. All authors have read, revised critically and approved the final submitted manuscript, and agreed to be accountable for the content of the work.

## Conflict of Interest Statement

The authors declare that the research was conducted in the absence of any commercial or financial relationships that could be construed as a potential conflict of interest.

## Footnotes

1 www.fmrib.ox.ac.uk/fsl

2 http://ric.uthscsa.edu/mango/

## Abbreviations

AS: ankle stimulation
BA: Brodmann area
BOLD: blood oxygenation level-dependent
EEG: electroencephalography
EPI: echo-planar imaging
FIR: finite impulse response
fMRI: functional magnetic resonance imaging
FWE: family-wise error
FWHM: full width at half maximum
GLM: general linear model
HRF: hemodynamic response function
HS: heel stimulation
IPL: inferior parietal lobule
M1: primary motor cortex
MNI: Montreal Neurological Institute
MPRAGE: magnetization-prepared rapid acquisition with gradient echo
MRI: magnetic resonance imaging
PC: principal component
PMC: premotor cortex
PMRF: pontomedullary reticular formation
PRF: pontine reticular formation
ROI: region of interest
S1: primary somatosensory cortex
S2: secondary somatosensory cortex
SD: standard deviation
SMC: sensorimotor cortex
SPL: superior parietal lobule
VAS: visual analog scale.
