# Supplementary material for: Differential Effects of Sustained Manual Pressure Stimulation According to Site of Action
Source: Front Neurosci. 2019 Jul 17;13:722. doi: 10.3389/fnins.2019.00722 (PMC6650750; doi:10.3389/fnins.2019.00722)
Supplement: Supplementary file 1 [file Table_1.docx]

Supplementary Material

# Supplementary Table

**Supplementary Table S1.** List of significant F-test clusters in Contrast 3 (HS + AS) – overall stimulation effect.

| Cluster Index | Subsystem (Heel, Ankle) | Anatomical atlas labels^a^ | Cytoarchitectonic atlas labels^a^ | Volume [cm^3^] | Cluster *p* | Z_max_ | Z_max_ MNI coordinates [x,y,z (mm)] |
| --- | --- | --- | --- | --- | --- | --- | --- |
| 1 | P, P | 15.5% L Frontal Pole  11.4% L Parietal Operculum C  10.8% L Angular G  9.0% L Supramarginal G, p. d.  7.5% L Central Opercular C  5.9% L Inferior Frontal G, pars triangularis  5.8% L Supramarginal G, a. d. | 14.6% L Broca's Area BA45  10.1% L Inferior Parietal Lobule PF  9.6% L Secondary Somatosensory C / Parietal Operculum OP1  8.8% L Inferior Parietal Lobule PFm  8.6% L Inferior Parietal Lobule PGa  6.3% L Inferior Parietal Lobule PFcm | 47.10 | <0.001 | 8.21 | -50, 24, -10 |
| 2 | N, N | 23.2% R Lateral Occipital C, s. d.  21.2% R Lingual G  16.8% L Lateral Occipital C, s. d.  7.3% R Occipital Pole  5.9% R Cuneal C  5.5% L Cuneal C | 19.9% R Visual C V2 BA18  6.9% R Visual C V1 BA17  6.1% L Superior Parietal Lobule 7A  6.1% L Visual C V2 BA18 | 39.51 | <0.001 | 8.04 | -16, -88, 32 |
| 3 | P, P | 20.8% L Postcentral G  16.6% L Superior Frontal G  14.8% L Precentral G  9.3% R Precuneous C  9.2% L Precuneous C  7.2% L Superior Parietal Lobule  5.7% R Superior Parietal Lobule | 21.3% L Premotor C BA6  14.7% L Primary Motor C BA4a  11.2% L Superior Parietal Lobule 5L  10.1% R Superior Parietal Lobule 5L  9.0% L Superior Parietal Lobule 7A  6.1%L Superior Parietal Lobule 5M | 33.38 | <0.001 | 8.79 | -14, -40, 70 |
| 4 | P, P | 20.9% R Supramarginal G, p. d.  16.3% R Parietal Operculum C  13.2% R Angular G  12.7% R Supramarginal G, a. d.  10.1% R Superior Temporal G, p. d.  9.4% R Middle Temporal G, temporooccipital part  7.9% R Planum Temporale | 22.4% R Inferior Parietal Lobule PF  17.6% R Inferior Parietal Lobule PGa  13.2% R Secondary Somatosensory C / Parietal Operculum OP1  12.0% R Inferior Parietal Lobule PFm  10.8% R Inferior Parietal Lobule PFcm | 28.24 | <0.001 | 8.21 | 56, -50, 10 |
| 5 | N, N | 63.7% R Precentral G  36.0% R Postcentral G | 34.3% R Premotor C BA6  14.3% R Primary Somatosensory C BA3b  12.7% R Primary Motor C BA4a  11.2% R Primary Somatosensory C BA1  8.9% R Primary Motor C BA4p  5.3% R Primary Somatosensory C BA3a | 25.13 | <0.001 | 8.21 | 42, -14, 36 |
| 6 | N, N | 54.8% L Precentral G  45.2% L Postcentral G | 24.3% L Premotor C BA6  17.7% L Primary Somatosensory C BA1  14.3% L Primary Somatosensory C BA3b  12.8% L Primary Motor C BA4p  10.9% L Primary Motor C BA4a  7.9% L Primary Somatosensory C BA3a | 19.50 | <0.001 | 8.21 | -50, -8, 34 |
| 7 | N, N | 62.0% L Lingual G  13.7% L Cerebellum V  11.0% L Precuneous C  10.4% L Occipital Fusiform G  9.7% L Cerebellum VI  5.4% L Temporal Occipital Fusiform C | 26.2% L Visual C V2 BA18  20.9% L Visual C V4  11.1% L Visual C V1 BA17  9.5% L Visual C V3V | 14.12 | <0.001 | 8.04 | -16, -58, -10 |
| 8 | P, P | 50.9% R Thalamus  43.8% L Thalamus | N/A | 5.06 | <0.001 | 7.84 | 8, -16, 12 |
| 9 | N, N | 95.1% R Lateral Occipital C, i. d. | 54.7% R Visual C V5  14.2% R Visual C V4  12.8% R Inferior Parietal Lobule PGp | 2.75 | <0.001 | 6.72 | 44, -78, 6 |
| 10 | P, P | 54.2% R Cingulate G, p. d.  24.3% L Cingulate G, p. d.  15.0% R Cingulate G, a. d.  6.5% L Cingulate G, a. d. | N/A | 0.86 | <0.001 | 6.63 | 4, -16, 32 |
| 11 | S, P | 99.0% R Central Opercular C | 75.0% R Secondary Somatosensory C / Parietal Operculum OP4  13.5% R Secondary Somatosensory C / Parietal Operculum OP3  7.3% R Broca's Area BA44 | 0.77 | <0.001 | 7.17 | 50, 2, 6 |
| 12 | S, P | 33.3% R Frontal Orbital C  28.3% R Temporal Pole  21.7% R Inferior Frontal G, pars triangularis  11.7% R Frontal Operculum C | 43.3% R Broca's Area BA45  33.3% R Primary Auditory C TE1.2 | 0.48 | <0.001 | 7.00 | 50, 18, -8 |
| 13 | N, N | 89.7% L Lateral Occipital C, superior division  10.3% L Superior Parietal Lobule | 100.0% L Superior Parietal Lobule 7A | 0.46 | <0.001 | 5.73 | -28, -64, 58 |
| 14 | N, N | 79.1% R Occipital Pole  20.9% R Lateral Occipital C, inferior division | 86.0% R Visual C V3V  9.3% R Visual C V4 | 0.34 | <0.001 | 5.70 | 32, -92, 0 |
| 15 | N, N | 81.4% L Middle Frontal G  18.6% L Superior Frontal G | 32.6% L Premotor C BA6 | 0.34 | <0.001 | 7.02 | -30, 16, 60 |
| 16 | P, P | 79.5% L Cingulate G, a.d.  20.5% L SMA | 64.1% L Premotor C BA6 | 0.31 | <0.001 | 6.15 | -10, -4, 40 |
| 17 | P, P | 54.8% R Cingulate G, p.d.  25.8% R Precuneous C  19.4% R Precentral G | 96.8% R Superior Parietal Lobule 5Ci | 0.25 | <0.001 | 5.70 | 12, -30, 42 |
| 18 | N, N | 100.0% R Hippocampus  20.0% R Parahippocampal G, a. d. | 80.0% R Hippocampus Cornu Ammonis  20.0% R Hippocampus Subiculum | 0.24 | <0.001 | 5.83 | 26, -16, -16 |
| 19 | S, P | 48.3% R Frontal Orbital C  41.4% R Insular C  10.3% R Frontal Operculum C | N/A | 0.23 | <0.001 | 5.42 | 32, 26, 0 |
| 20 | P, P | 92.6% Brain-Stem  7.4% L Thalamus | N/A | 0.22 | <0.001 | 6.04 | -8, -28, -6 |
| 21 | S, N | 87.0% L Paracingulate G  13.0% R Paracingulate G | N/A | 0.18 | <0.001 | 5.72 | -6, 44, 22 |
| 22 | S, P | 100.0% Brain-Stem | N/A | 0.18 | <0.001 | 5.93 | -8, -30, -34 |
| 23 | N, N | 100.0% R Lingual G | 85.7% R Visual C V2 BA18  14.3% R Visual C V1 BA17 | 0.17 | <0.001 | 5.31 | 8, -82, -10 |
| 24 | N, N | 100.0% L Frontal Pole | N/A | 0.16 | <0.001 | 5.69 | -18, 54, 30 |
| 25 | N, N | 100.0% L Frontal Pole | N/A | 0.14 | 0.001 | 5.67 | -18, 62, 22 |
| 26 | S, P | 100.0% R Inferior Frontal G, pars opercularis | 64.7% R Broca's Area BA45  35.3% R Broca's Area BA44 | 0.14 | 0.001 | 5.47 | 54, 20, 16 |
| 27 | N, N | 100.0% L Middle Frontal G | 18.8% L Premotor C BA6 | 0.13 | 0.001 | 5.45 | -42, 12, 54 |
| 28 | P, P | 100.0% R Cingulate G, p. d. | N/A | 0.12 | 0.001 | 5.63 | 6, -40, 24 |
| 29 | P, P | 100.0% R Cerebellum VIIIb  7.1% Brain-Stem | N/A | 0.11 | 0.001 | 6.17 | 18, -46, -54 |
| 30 | S, P | 100.0% L Insular C | 25.0% L Broca's area BA44 | 0.06 | 0.002 | 5.37 | -36, 22, -2 |
| Abbreviations: a. d. – anterior division; C – cortex; BA – Brodmann area; G – gyrus; i. d. – inferior division; L – left; N – task-negative; N/A – not available; MNI – Montréal Neurological Institute; P – task-positive; p. d. – posterior division; R – right; S – sustained task-positive; s. d. – superior division; SMA – supplementary motor area (also juxtapositional lobule cortex); Z_max_ – maximum Z score.  ^a^) Anatomical and cytoarchitectonic labels are provided including the proportion of labeled voxels. Only labels consisting at least 5% of activated voxels are provided. Note that cerebellar labels may overlap with cortical labels and that cytoarchitectonic labels do not cover the whole brain. | | | | | | | |
