# Supplementary material for: Differential Effects of Sustained Manual Pressure Stimulation According to Site of Action
Source: Front Neurosci. 2019 Jul 17;13:722. doi: 10.3389/fnins.2019.00722 (PMC6650750; doi:10.3389/fnins.2019.00722)
Supplement: Supplementary file 2 [file Data_Sheet_1.docx]

Supplementary Material

# Supplementary Figure

**Supplementary Figure S1.** Hierarchical clustering of group-wise BOLD signal responses. Dendrograms illustrate agglomerative hierarchical clustering of correlation coefficients of BOLD signal responses in significant clusters obtained from Contrast 3 (AS + HS, i.e., mean pooled response to both heel and ankle stimulation). On the left, clusters are grouped according to distance of correlation coefficients in Heel condition, whereas on the right, responses were clustered in Ankle condition. Abscissa represents the Euclidean distance between clusters, whereas ordinate represents correlation coefficient vectors, one per a significant cluster in Contrast 3. Colours distinguish clusters as indicated by Caliński-Harabasz criterion (Caliński and Harabasz 1974).

# References

Caliński, T., and Harabasz, J. (1974). A dendrite method for cluster analysis. *Commun Stat Theory Methods* 3, 1–27.
